# From Theory to Practice in Inclusive Education: Effects of a Consecutive Training Pathway in Physical Activity and Sport Sciences Students

**DOI:** 10.3390/bs16030360

**Published:** 2026-03-04

**Authors:** Bingen Marcos-Rivero, Jon Ortuondo, Matías Henríquez, María Isabel Cornejo, Javier Yanci

**Affiliations:** 1 AKTIBOki: Research Group in Physical Activity, Exercise, and Sport, Department of Physical and Sports Education, Faculty of Education and Sport, University of the Basque Country (UPV/EHU), 01007 Vitoria-Gasteiz, Spain; jortuondo@bam.eus (J.O.); javier.yanci@ehu.eus (J.Y.); 2 Society, Sports and Physical Exercise Research Group (GIKAFIT), Physical Education and Sport Department, Faculty of Education and Sport, University of the Basque Country (UPV/EHU), 01007 Vitoria-Gasteiz, Spain; 3 Department of Music Expression, Plastic Arts and Body Expression, Begoñako Andra Mari University School of Education (BAM), 48160 Derio, Spain; 4 Escuela de Kinesiología, Facultad de Salud, Universidad Santo Tomas, Av. Ejército Libertador 146, Santiago 8370003, Chile; mhenriquez24@santotomas.cl (M.H.); mcornejo20@santotomas.cl (M.I.C.)

**Keywords:** inclusive practice, higher education, physical education, teacher education, professional development

## Abstract

**Introduction**: Despite the growing emphasis on inclusion, evidence on the longitudinal effects of consecutive training pathways in physical activity remains limited, particularly within initial university-level education. **Objective**: To assess the impact of a consecutive inclusive physical activity training pathway on the academic and professional development of Physical Activity and Sport Sciences students, focusing on academic self-concept, perceptions of educational inclusion, and evaluations of theoretical and practical training. **Method**: A longitudinal, quantitative repeated-measures design was employed across one full academic year. University students responded to questionnaires at three time points (R1, R2, and R3), corresponding to key phases of the training pathway. Within-subject analyses were conducted to examine changes over time throughout the complete course sequence. **Results**: Significant reductions were observed in academic self-concept, perceived academic performance, and academic self-efficacy across the training pathway. Concurrently, significant improvements were found in key dimensions of inclusive practice, particularly in methodology, supports, and community participation, whereas the conception of diversity remained stable. Perceived adequacy of theoretical training increased progressively over time, while perceived adequacy of practical training improved following the first course and subsequently stabilized. **Conclusions**: The training pathway positively influences students’ preparedness for inclusive education. However, the findings suggest the need to strengthen structured practical experiences that facilitate the transfer of learning to professional practice.

## 1. Introduction

Physical activity (PA) is a fundamental determinant of health and quality of life, with well-documented benefits across physical, psychological, and social domains (Marquez et al., 2020; Warburton et al., 2006). For individuals with disabilities, PA is particularly relevant, as regular participation is associated with improvements in functional capacity, autonomy, and community participation (Hassett et al., 2024; Hutzler et al., 2013). Despite these benefits, evidence consistently indicates that this population engages in lower levels of PA compared with those without disabilities (Carr et al., 2025a, 2025b; Noetel et al., 2025). This disparity has been attributed to personal, social, and structural barriers that restrict access to and sustained participation in exercise, PA, or Physical Education (PE) programs (J. Haegele et al., 2018; Liu et al., 2025; MacEachern et al., 2021). This scenario underscores the need to promote inclusive environments (Richards, 2025) and to reduce inequalities in PA participation (Carr et al., 2025a, 2025b), favoring participation of people with disabilities. PE represents a particularly conducive context for the development of inclusive practices due to its social, motor, and experiential nature, which facilitates interaction and cooperation among students with and without disabilities (J. A. Haegele & Sutherland, 2015; Qi & Ha, 2012).

In this regard, the literature emphasizes that inclusion in PE cannot be limited solely to access to motor practice. A sense of belonging, acceptance, and recognition is essential to the educational experience of students with disabilities, whose participation and well-being largely depend on the quality of their social interactions and perceived value within the class context (BeyazoĞlu & Haegele, 2025; Wilson et al., 2020b). The role of future professionals in PA and Sport Sciences, as well as PE teachers, is decisive in ensuring the full participation of students with and without disabilities across educational, sport, and community contexts (Liu et al., 2025; Wei & Chang, 2024; Wilson et al., 2020b). Research in PE teacher education indicates that pre-service teachers enter training with beliefs and pedagogical orientations shaped by prior experiences, which can influence the adoption of inclusive practices if not critically examined (Curtner-Smith et al., 2025).

Consequently, the ability to design and adapt tasks, manage supports, and create accessible learning environments constitutes a core professional competence for promoting PA participation among students with greater support needs, particularly given that inclusivity depends on task modifications, resource availability, and interprofessional coordination (Wilson et al., 2020b). Initial training in inclusion should therefore provide future PE professionals with a strong pedagogical foundation and structured opportunities to integrate theory and practice across diverse contexts (Brown et al., 2019; Iloanya, 2019). However, recent evidence suggests persistent gaps in this preparation. For example, Salin et al. (2025) showed that, although pre-service teachers’ teaching self-efficacy increases throughout undergraduate training, substantial limitations remain in addressing the requirements of students with special educational needs. Collectively, these findings suggest that current initial teacher education programs may not be adequately equipping students with the competencies required for effective inclusive education.

Research in inclusive education suggests that teachers’ capacity to foster inclusion depends on an integrated set of competencies that combine specialized knowledge, pedagogical skills, and positive attitudes toward diversity (Letzel-Alt & Pozas, 2025; Tveitnes et al., 2025; Vantieghem et al., 2023). In addition, inclusive competence is not limited to the possession of theoretical knowledge, but also requires self-efficacy, decision-making capacity, and collaboration with colleagues and families (Vantieghem et al., 2023). Consistent with this view, many teachers perceive gaps in their professional knowledge when addressing complex educational needs, thereby reinforcing the need to strengthen both initial and continuing teacher education in inclusive competencies (Tveitnes et al., 2025). From this perspective, the present study acknowledges a participation-oriented approach consistent with bio-psycho-social models of inclusion, in which engagement in physical activity is understood as a function of individual functioning, contextual supports, and opportunities for meaningful participation.

Despite conceptual advances in inclusive PA and PE, empirical evidence on the effects of longitudinal training programs for university students in PA and Sport Sciences (i.e., also future PE teachers) remains limited. Most available studies focus on short-term training initiatives (Alhumaid et al., 2021; Alhumaid, 2023), standalone courses (Jiménez-Monteagudo et al., 2023; Reina et al., 2019b), or isolated experiences (Kwon et al., 2022), which makes it difficult to understand how the acquisition of inclusive competencies evolves when training is delivered progressively over an extended academic period. Evaluating a consecutive training pathway across undergraduate education could provide a more robust perspective on the cumulative effects of initial teacher education and on the competency level with which students enter the profession. Therefore, the present study aimed to examine the impact of a consecutive training pathway, composed of the courses “*Inclusive Physical Activity*” and “*Physical Activity for the Health of People with Disabilities*,” on the academic and professional development of students enrolled in the Bachelor’s Degree in PA and Sport Sciences. Specifically, the study analyzed how this training process influenced academic self-concept, perceptions of educational inclusion and attention to diversity, and evaluations of the adequacy of the theoretical and practical training received to address inclusion in professional contexts related to physical activity and sport.

## 2. Materials and Methods

### 2.1. Research Design

A longitudinal, quantitative, repeated-measures design was employed to analyze changes in students’ perceptions of their theoretical and practical training in inclusive PE across a consecutive training pathway delivered over one academic year. Data were collected at three time points (R1, R2, and R3), corresponding to key phases of the training process: prior to the start of the compulsory course (R1), at the end of the first semester following completion of this course (R2), and at the end of the second semester after completion of the elective course *Physical Activity for the Health of People with Disabilities* (R3). This design enabled the examination of within-subject changes over time in relation to the progressive acquisition of inclusion-related training.

### 2.2. Participants

A total of 31 undergraduate students (13 men and 18 women) enrolled in the fourth year of the Bachelor’s Degree in Sport and Exercise Sciences (SES) at a Spanish public university participated in the study. All participants completed the compulsory course *Inclusive PA* (first semester) and the elective course *PA for the Health of People with Disabilities* (second semester) during one academic year. The inclusion criteria were as follows: (1) enrollment in both courses (compulsory and elective), (2) attendance of at least 80% of the scheduled sessions in both courses, (3) continuous participation in the courses from the beginning to the end of the study, and (4) completion of the pretest and posttest assessments. Given the longitudinal nature of the study across a full academic year and the requirement that participants be enrolled in both courses, including the elective course offered in the second semester, the final sample corresponds to the available cohort meeting these criteria and represents a substantial proportion of the eligible population. Institutional authorization was obtained from the university, the corresponding department, and the faculty responsible for the courses. The study was conducted in accordance with the guidelines of the World Medical Association (2025) and received approval from the Ethics Committee for Research with Human Beings of the University of the Basque Country (UPV/EHU) (CEISH M10/2023/280). In addition, the project was selected for funding under the UPV/EHU Educational Innovation Projects Call HPB/PIE i3lab (2025–2026).

### 2.3. Procedure

The study was conducted over a full academic year using a quasi-experimental repeated-measures design (pretest–posttest), structured around three assessment points. During the first semester, participants completed the compulsory course *Inclusive PA*, and during the second semester, they completed the elective course *PA for the Health of People with Disabilities*, both undertaken by all participants during the fourth year of the Bachelor’s Degree in PA and Sport Sciences. Data were collected at three time points: prior to the start of the first course (R1), at the end of the first semester following completion of Inclusive PA (R2), and at the end of the second semester after completion of PA for the Health of People with Disabilities (R3). Both courses were delivered by faculty members with expertise in disability, inclusion, and PA. The Inclusive PA course (6 ECTS) followed a theoretical–practical approach, combining lectures, seminars, and classroom-based practical sessions aimed at experiential engagement with inclusive situations. In contrast, the PA for the Health of People with Disabilities course (4.5 ECTS) adopted a predominantly theoretical approach, based on lectures, case analyses, and guided discussions, with reflective and group activities conducted in simulated contexts without direct practical experience. Across both courses, assessment included continuous coursework (practical assignments), a final project, and a written examination. Course content addressed foundational concepts of disability and inclusion, physical activity and health, and the design of inclusive or health-oriented intervention programs for people with disabilities.

### 2.4. Compulsory Course: Inclusive Physical Activity

The intervention was implemented within the compulsory course *Inclusive PA*, delivered during the first semester. The course comprises 60 contact hours (30 theoretical sessions, 10 seminar hours, and 20 practical sessions). Its primary objective is to provide students with conceptual and applied foundations in inclusive PE and sport, fostering competencies to design and implement activities that ensure the participation of all individuals, with and without disabilities, across PA and PE contexts. Teaching followed a theoretical–practical approach, combining interactive lectures with seminars and classroom-based practical sessions aimed to encourage reflection and experiential engagement with inclusive situations. While theoretical sessions included larger groups (20–90 students), seminars and practical sessions were conducted in smaller subgroups (20–30 students) to facilitate active participation. Course content was structured into four main blocks: (1) foundations of inclusive PA and types of disability; (2) inclusion in educational contexts and PE; (3) adapted sport and sport classification systems; and (4) inclusive physical activities in leisure contexts.

### 2.5. Elective Course: Physical Activity for the Health of People with Disabilities

The second intervention was implemented within the elective course *PA for the Health of People with Disabilities*, delivered during the second semester. The course aimed to provide students with specialized knowledge on the effects of physical exercise on the health of people with disabilities, as well as competencies to design and analyze PA programs intended to improve health outcomes in this population. Instruction followed a predominantly theoretical approach, consisting of lectures supported by audiovisual materials, case analyses, and guided discussions. Although reflective activities and group work were encouraged, these were conducted in a simulated context, without direct practical experiences. Student assessment included the submission of 10 practical assignments, the development and oral defense of a final project focused on physical exercise programs for people with disabilities, and a written examination. Course content was structured into four blocks: (1) foundations of PA, health, and disability; (2) organization of PA for health and professional roles; (3) effects of exercise according to type of disability (physical, sensory, psychological, and intellectual), although course content is organized according to general disability typologies for pedagogical purposes, the instructional approach emphasizes individual functioning, activity limitations, participation, and contextual factors influencing engagement in physical activity settings; and (4) design of intervention programs aimed at improving health.

### 2.6. Measures

Data collection was conducted at three points (R1, R2, and R3) throughout the academic year. Three main instruments were used: the Academic Self-Concept Scale (ASCS), the Questionnaire for the Evaluation of Teacher Training for Inclusion (CEFI-R), and two ad hoc items assessing students’ perceptions of their theoretical and practical training in inclusion. To assess university students’ academic self-concept across the academic year, the ASCS, validated by Schmidt et al. (2008), was employed. This instrument assesses academic self-concept across three dimensions: academic self-concept, academic performance, and academic self-efficacy. The scale consists of 14 items rated on a 5-point Likert-type scale (1 = strongly disagree; 5 = strongly agree).

To assess students’ training and perceptions regarding educational inclusion and attention to diversity, the CEFI-R was used (González-Gil et al., 2019). The instrument comprises 19 items distributed across four dimensions: Conception of Diversity (5 items), Methodology (5 items), Supports (4 items), and Community Participation (5 items). The Conception of Diversity dimension assesses teachers’ beliefs, attitudes, and values toward diversity and inclusive education. The Methodology dimension refers to the adaptation of teaching strategies, curriculum design, assessment, materials, and communication to address diverse learners. The Supports dimension focuses on the interpretation and organization of educational supports, including collaboration among teachers and the role of support professionals within inclusive settings. Finally, the Community Participation dimension evaluates the degree of collaboration and involvement of families, professionals, and community resources in the educational process. Responses were recorded on a four-point Likert scale (1 = strongly disagree; 4 = strongly agree). In the last three dimensions, higher scores indicate more positive evaluations, whereas in the Conception of Diversity dimension, the scale is reverse-coded, such that lower values reflect a more favorable conception of diversity. As a complement to the ASCS and CEFI-R questionnaires, two quantitative items were included to assess students’ perceptions of their theoretical and practical training in inclusion. The items were: “Do you consider your theoretical training sufficient to address inclusion in your future professional practice?” and “Do you consider your practical training sufficient to address inclusion in your future professional practice?” Responses were recorded on an eight-point Likert scale (1 = minimal perceived level of sufficiency; 8 = maximum perceived level).

### 2.7. Statistical Analysis

Data are presented as mean ± standard deviation. Normality was assessed using the Shapiro–Wilk test, and homogeneity of variances was examined with Levene’s test. As most variables did not meet the assumptions of normality, nonparametric procedures were applied. Specifically, a repeated-measures analysis of variance based on Kendall’s W was conducted, with post hoc pairwise comparisons adjusted using the Holm correction method. Effect size, to estimate the magnitude of pairwise mean differences, was calculated using the rank-biserial correlation (*r_b_*), interpreted as <0.10 (very small), 0.10–0.29 (small), 0.30–0.49 (moderate), and ≥0.50 (large) (López-Martín & Ardura, 2023). Statistical analyses were performed using JASP software (JASP for macOS, version 0.95.4, Amsterdam, The Netherlands). The level of statistical significance was set at *p* < 0.05.

## 3. Results

Table 1 presents the results for the ASCS across the three assessment points: R1 (prior to *Inclusive PA* course), R2 (post-Inclusive PA and pre-PA for the Health of People with Disabilities), and R3 (post-*PA for the Health of People with Disabilities*). Overall, after completing both courses, significant reductions were observed between R1 and R3 in academic self-concept (*r_b_* = 0.95, *p* < 0.001, large), perceived academic performance (*r_b_* = 0.85, *p* < 0.001, large), and academic self-efficacy (*r_b_* = 0.96, *p* < 0.001, large). Regarding changes between R1 and R2, significant reductions were found in academic self-concept (*r_b_* = 0.55, *p* < 0.05, large) and perceived academic performance (*r_b_* = 0.64, *p* < 0.05). Finally, after completing the elective course, significant reductions between R2 and R3 were observed in academic self-concept (*r_b_* = 0.98, *p* < 0.001, large), perceived academic performance (*r_b_* = 0.83, *p* < 0.01), and academic self-efficacy (*r_b_* = 0.99, *p* < 0.001, large).

Table 2 presents the CEFI-R results across the three assessment points. Overall, after completion of the courses, significant improvements were observed between R1 and R3 in several dimensions related to inclusive practice, whereas the Conception of Diversity dimension remained stable throughout the training process. In the Methodology dimension, significant increases were found between R1 and R3 (*r_b_* = −0.84, *p* < 0.001, large) and between R1 and R2 (*r_b_* = −0.96, *p* < 0.001, large). Similarly, in the Supports dimension, significant increases between R1 and R3 (*r_b_* = −0.45, *p* < 0.05, moderate) and between R1 and R2 (*r_b_* = −0.48, *p* < 0.05) were found, while no further changes were observed between R2 and R3. Finally, the Community Participation dimension showed significant differences across all comparisons: between R1 and R3 (*r_b_* = −0.50, *p* < 0.001, large), between R1 and R2 (*r_b_* = −0.68, *p* < 0.05, large), and between R2 and R3 (*r_b_* = −0.23, *p* < 0.05, small). In contrast, the Conception of Diversity dimension did not show significant differences across the three assessment points (Kendall’s W = 0.01, *p* > 0.005).

**Figure 1:**
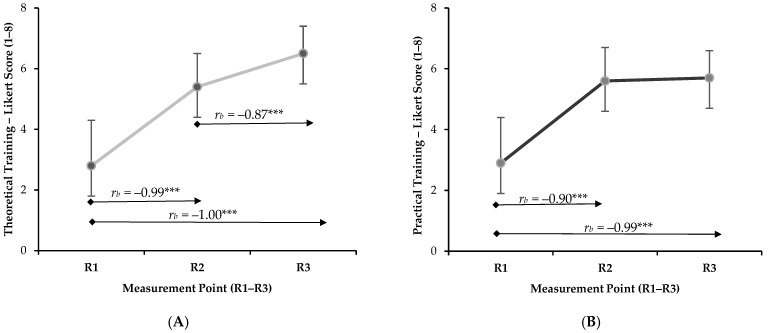
Evolution of perceived theoretical and practical preparedness across the training pathway: (**A**) perceived theoretical preparedness across the three measurement points (R1–R3); (**B**) perceived practical preparedness across the three measurement points (R1–R3). **Note.** R1 = prior to Inclusive Physical Activity; R2 = after completion of Inclusive Physical Activity and before commencement of Physical Activity for the Health of People with Disabilities; R3 = after completion of Physical Activity for the Health of People with Disabilities. Higher scores indicate greater perceived preparedness. Kendall’s W-based repeated-measures analysis with post hoc pairwise comparisons adjusted using the Holm correction. * *p* < 0.05; ** *p* < 0.01; *** *p* < 0.001.

Perceptions of theoretical training increased progressively across the three assessment points, rising from R1 (2.8 ± 1.5) to R2 (5.4 ± 1.1) and reaching the highest value at R3 (6.5 ± 0.9). In contrast, perceptions of practical training increased markedly after the first course, from R1 (2.9 ± 1.8) to R2 (5.6 ± 1.4), and then remained stable at R3 (5.7 ± 1.1). Figure 1 illustrates the effects of the intervention on students’ perceptions of the training received.

Overall, completion of the first course was associated with significant improvements in both theoretical and practical training perceptions. However, after the second course, additional progress was observed only in theoretical training, whereas perceptions of practical training remained stable. Theoretical training showed significant increases between R1 and R3 (*r_b_* = −1.00, *p* < 0.001, large) and between R1 and R2 (*r_b_* = −0.99, *p* < 0.001, large), and also maintained significant differences between R2 and R3 (*r_b_* = −0.87, *p* < 0.001, large), evidencing a sustained improvement throughout the training process. In contrast, practical training also showed significant increases between R1 and R3 (*r_b_* = −0.99, *p* < 0.001, large) and between R1 and R2 (*r_b_* = −0.90, *p* < 0.001, large). No subsequent differences were found between R2 and R3, indicating that perceptions of practical training improved markedly after the first course and remained stable thereafter.

## 4. Discussion

This study aimed to analyze the impact of a consecutive training pathway, composed of two courses of PA in people with disabilities, on the academic and professional development of students enrolled in the Bachelor’s Degree in Physical Activity and Sport Sciences. Importantly, within the analyzed curriculum, these two courses represent the entirety of the compulsory and elective training specifically related to disability and inclusion. Specifically, the study examined how this training process influenced students’ academic self-concept, their perceptions of educational inclusion and attention to diversity, and their evaluation of the perceived adequacy of the theoretical and practical preparation received to competently address inclusion in future professional contexts related to PE or PA. While previous research highlights the necessity of including inclusion-focused content in PE- and PA-related curricula (Marcos-Rivero et al., 2025; Piletic & Davis, 2010), it is equally important to assess whether such training effectively prepares students to meet the real demands of professional practice (Jiménez-Monteagudo et al., 2023; Reina et al., 2019a). The present findings contribute to this discussion by adopting a longitudinal perspective across the full sequence of inclusion-related training within the degree program.

Analyzing the evolution of academic self-concept among university students is essential for understanding how they interpret their learning processes and professional preparation (Jackman et al., 2011; Rosman et al., 2020). In the present study, the results reveal a progressive and significant reduction in academic self-concept and perceived academic performance throughout the training pathway, as well as a decline in academic self-efficacy during the later stages. This pattern has been reported in previous research, which suggests that as disciplinary knowledge increases and understanding of professional complexity deepens, students tend to engage in more critical self-assessments of their own competencies (Symes et al., 2023). Within the field of training in inclusion and disability, this tendency may be further accentuated by increased awareness of the real challenges associated with professional practice and of the responsibility inherent in inclusive intervention. This interpretation aligns with the concept of the *reality shock* (Botha & Rens, 2018; Veenman, 1984; Voss & Kunter, 2020), which describes how progressive confrontation with the complexity and real demands of professional practice leads to a critical reassessment of one’s own capabilities and expectations. This process may result in a decline in perceived self-concept or self-efficacy despite increases in knowledge and objective competence (Veenman, 1984). From this perspective, the observed decrease should not be interpreted as a negative effect of the training, but rather as a potential indicator of academic and professional maturation. This interpretation is consistent with models that conceptualize academic self-concept as a dynamic construct, sensitive to formative experiences, and subject to adjustment as academic demands and understanding of one’s own performance increase (Marsh & Martin, 2011). Future research should explore moderating factors, such as the nature of practical experiences or structured reflection, that may shape this trajectory.

With respect to perceptions of preparedness for inclusive practice, the findings indicate a differentiated pattern across dimensions. Consistent with previous studies reporting limited perceived preparedness among future PE teachers (Marcos-Rivero et al., 2025; Wilson et al., 2020a), the present results suggest that inclusion-focused training can modify certain competence-related perceptions, though not uniformly. In this context, it is necessary to examine the effects of inclusion-focused training throughout the SES degree program (Jiménez-Monteagudo et al., 2023; McCracken et al., 2023). Within this framework, the results of the present study suggest that specific training in inclusion across the SES degree contributes to modifying certain perceptions related to teaching competence, although these changes do not occur uniformly across all assessed dimensions. In particular, the improvements observed in the Methodology and Supports dimensions may be interpreted in light of the training content of the *Inclusive PA* course, which prioritizes explicit work on methodological strategies, adaptations, and supports in PE. These elements have been identified as key components in the development of inclusive teaching competence (Molina Garuz et al., 2023). The absence of additional improvements following the second course may indicate that, once a solid conceptual framework has been acquired, initial theoretical training is sufficient to consolidate this type of knowledge. In contrast, the progressive evolution observed in the Community Participation dimension appears to reinforce the notion that this construct is enhanced when inclusion is addressed in a transversal and sustained manner throughout the curriculum, rather than through isolated content blocks. By contrast, the lack of change in the Conception of Diversity dimension may be explained by students’ already high level of prior awareness of these issues, which is consistent with the current social and educational context in which discourses on diversity and inclusion are widely present (Marcos-Rivero et al., 2025). This high baseline may have limited the observable margin for improvement, despite the fact that this dimension was explicitly addressed during the training process. Taken together, these findings invite reflection on the need to differentiate between dimensions of a more cognitive and procedural nature, which may be amenable to improvement through specific theoretical training, and those linked to the practical enactment of inclusion. The latter may require the incorporation of specific pedagogical approaches and models that facilitate the transfer of knowledge into professional practice through meaningful learning experiences, particularly in initial teacher education contexts, where such transfer has been shown not to occur automatically and to require processes of pedagogical recontextualization and experiential engagement (Backman et al., 2023).

To date, the existing evidence on inclusion training within degree programs that qualify graduates to teach PE remains inconclusive, as some studies have adopted predominantly theoretical approaches (Roldan & Reina, 2021), while others have prioritized practical experiences (Jiménez-Monteagudo et al., 2023), observing positive effects on different training-related variables in both cases. However, beyond these effects, the question remains as to whether such training enables future teachers to perceive themselves as sufficiently competent, both theoretically and practically, to meet the real demands of inclusive education. In this regard, the results of the present study revealed a progressive and significant increase in perceptions of theoretical training throughout the entire training pathway (R1–R2, R2–R3, and R1–R3). In contrast, perceptions of practical training improved significantly after the first course (R1–R2) and subsequently remained stable, with no further changes observed between R2 and R3. It is noteworthy that, unlike findings reported in other studies in which graduates do not perceive themselves as sufficiently prepared (Marcos-Rivero et al., 2025; Wilson et al., 2020a), students in the present study reported feeling more competent in educational inclusion after completing the training pathway. Nevertheless, although the second course, characterized by a more theoretical focus, did not produce further changes in perceptions of practical preparation, it may be argued that incorporating a practical component into this course could help consolidate the knowledge acquired and further enhance future teachers’ practical readiness, despite the lower credit load of the elective course. In this regard, practical experiences are essential for the development of self-efficacy in inclusive education, as positive experiences have been shown to significantly increase future teachers’ confidence in organizing and managing inclusive classrooms (Weber & Greiner, 2019). Moreover, Pérez-Restrepo et al. (2025) emphasize the importance of experiencing pedagogical models during teacher education, as this allows future professionals to integrate theory and practice, reflect on their performance, and adapt different pedagogical approaches to their specific contexts. Consequently, the inclusion of additional practical experiences in the second course could strengthen students’ practical preparation and ensure they can apply their knowledge effectively in inclusive educational settings.

Taken together, these findings underscore the need to design training pathways that integrate theoretical content and practical experiences in a balanced manner, in order to strengthen perceived professional competence in alignment with the real demands of inclusive practice.

### Implications, Limitations, and Future Directions

From an applied perspective, the findings of this study provide relevant guidance for the design of training pathways within Bachelor’s degrees in PA and Sport Sciences. The results indicate that the inclusion of courses specifically focused on inclusion and disability contributes to improving students’ perceived preparedness, particularly at the theoretical level. However, the findings also highlight the need to complement conceptual training with experiential learning opportunities that facilitate the development of professional competencies transferable to real inclusive practice contexts. In this regard, the incorporation of situated practical activities, guided observation, service-learning, or supervised intervention experiences may promote greater alignment between inclusive discourse and professional action.

Nevertheless, several limitations of the present study should be acknowledged. First, the sample size was relatively small due to the longitudinal follow-up across a full academic year, the progressive loss of participants, and the elective nature of one of the courses analyzed, which limited enrollment numbers. Second, the training pathway evaluated was predominantly theoretical in nature, which restricts the extent to which conclusions can be drawn regarding the impact of more intensive practical experiences. In light of these limitations, future research should compare training pathways with differing levels of experiential density, examining not only perceptions and self-assessments but also indicators of applied performance and inclusive decision-making in real-world contexts. Given that initial teacher education may reproduce hegemonic frameworks that normalize segregating responses under an inclusive discourse (Pérez-Castejón et al., 2025), it is particularly important to adopt longitudinal designs that allow for the examination of whether training received during undergraduate education effectively translates into inclusive practices once students enter professional practice.

## 5. Conclusions

The present study provides relevant empirical evidence on the impact of a specific training pathway on perceptions of preparedness for inclusive education among future PE teachers and/or professionals in PA and sport who are graduates in SES, contributing to the literature through a longitudinal analysis of both theoretical and practical training. From an applied perspective, the findings highlight the need to design curricula that integrate theoretical content and practical experiences in a balanced manner, incorporating pedagogical approaches and models that promote the transfer of knowledge to real intervention contexts. In this regard, initial training in PE and SES is consolidated as a strategic context for fostering future inclusive practices, underscoring the importance of strengthening training experiences oriented toward the effective and sustainable application of inclusion in professional practice.

## Figures and Tables

**Table 1:** Effects of the intervention on the Academic Self-Concept Scale (ASCS) across three assessment points.

EAA	R1 M ± SD	R2 M ± SD	R3 M ± SD		Post Hoc		
				ANOVA Kendall’s W	R1–R2 *r_b_*	R1–R3 *r_b_*	R2–R3 *r_b_*
Dimension							
Academic Self-Concept	4.0 ± 0.6	3.8 ± 0.4	2.8 ± 0.5	0.55 ***	0.55 *	0.95 ***	0.98 ***
Perceived Performance	3.7 ± 0.8	3.3 ± 0.4	2.6 ± 0.4	0.33 ***	0.64 *	0.85 ***	0.83 **
Academic Self-Efficacy	4.2 ± 0.5	4.2 ± 0.5	2.9 ± 0.8	0.60 ***	–0.04	0.96 ***	0.99 ***

**Note**: R1 = before the Inclusive Physical Activity course; R2 = after the aforementioned course but before the Physical Activity for the Health of People with Disabilities course; R3 = after the latter course. In all dimensions, higher scores indicate more positive evaluations. * *p* < 0.05; ** *p* < 0.01; *** *p* < 0.001.

**Table 2:** Effects of the intervention on the dimensions of the Questionnaire for the Evaluation of Teacher Training for Inclusion (CEFI-R) across three assessment points.

CEFI-R	R1 M ± SD	R2 M ± SD	R3 M ± SD		Post Hoc		
				ANOVA Kendall’s W	R1–R2 *r_b_*	R1–R3 *r_b_*	R2–R3 *r_b_*
Dimension							
Diversity Conception	1.8 ± 0.4	1.7 ± 0.5	1.7 ± 0.5	0.01	0.24	0.02	–0.19
Methodology	2.5 ± 0.6	3.2 ± 0.4	3.1 ± 0.4	0.43 ***	–0.96 ***	–0.84 ***	0.07
Supports	3.0 ± 0.4	3.2 ± 0.4	3.2 ± 0.4	0.08 *	–0.48 *	–0.45 *	0.03
Community participation	3.3 ± 0.5	3.4 ± 0.4	3.5 ± 0.4	0.21 **	–0.68 *	–0.50 ***	–0.23 *

**Note**: R1 = before the Inclusive Physical Activity course; R2 = after the aforementioned course but before the Physical Activity for the Health of People with Disabilities course; R3 = after the latter course. In the Conception of Diversity dimension, lower scores indicate a more favorable perception of diversity, whereas in the remaining dimensions (Methodology, Supports, and Community Participation), higher scores indicate more positive evaluations. * *p* < 0.05; ** *p* < 0.01; *** *p* < 0.001.

## Data Availability

The data supporting the reported results are only available upon reasonable request from the corresponding author. Due to privacy restrictions, the data are not publicly available.

## Abbreviations

The following abbreviations are used in this manuscript:

ASCS	Academic Self-Concept Scale
CEFI-R	Questionnaire for the Evaluation of Teacher Training for Inclusion
ECTS	European Credit Transfer and Accumulation System
PA	Physical Activity
PE	Physical Education
SES	Sport and Exercise Sciences
